# Living on the Edge: Roe Deer (*Capreolus capreolus*) Density in the Margins of Its Geographical Range

**DOI:** 10.1371/journal.pone.0088459

**Published:** 2014-02-12

**Authors:** Ana M. Valente, Carlos Fonseca, Tiago A. Marques, João P. Santos, Rogério Rodrigues, Rita Tinoco Torres

**Affiliations:** 1 CESAM, Departamento de Biologia, Universidade de Aveiro, Aveiro, Portugal; 2 Universidade Lúrio, Campus de Marrere, Nampula, Mozambique; 3 Centre for Research into Ecological and Environmental Modelling, The Observatory, University of St Andrews, St Andrews, Scotland; 4 Instituto de Investigación en Recursos Cinegéticos, Ciudad Real, Spain; 5 Departamento de Conservação da Natureza e Florestas do Norte, Parque Florestal, Vila Real, Portugal; CNRS-Montpellier, France

## Abstract

Over the last decades roe deer (*Capreolus capreolus*) populations have increased in number and distribution throughout Europe. Such increases have profound impacts on ecosystems, both positive and negative. Therefore monitoring roe deer populations is essential for the appropriate management of this species, in order to achieve a balance between conservation and mitigation of the negative impacts. Despite being required for an effective management plan, the study of roe deer ecology in Portugal is at an early stage, and hence there is still a complete lack of knowledge of roe deer density within its known range. Distance sampling of pellet groups coupled with production and decay rates for pellet groups provided density estimates for roe deer in northeastern Portugal (Lombada National Hunting Area - LNHA, *Serra de Montesinho* – SM and *Serra da Nogueira* – SN; LNHA and SM located in Montesinho Natural Park). The estimated roe deer density using a stratified detection function was 1.23/100 ha for LNHA, 4.87/100 ha for SM and 4.25/100 ha in SN, with 95% confidence intervals (CI) of 0.68 to 2.21, 3.08 to 7.71 and 2.25 to 8.03, respectively. For the entire area, the estimated density was about 3.51/100 ha (95% CI - 2.26–5.45). This method can provide estimates of roe deer density, which will ultimately support management decisions. However, effective monitoring should be based on long-term studies that are able to detect population fluctuations. This study represents the initial phase of roe deer monitoring at the edge of its European range and intends to fill the gap in this species ecology, as the gathering of similar data over a number of years will provide the basis for stronger inferences. Monitoring should be continued, although the study area should be increased to evaluate the accuracy of estimates and assess the impact of management actions.

## Introduction

Over the past few decades ungulates have experienced an expansion throughout Europe both in number and distribution [1]. According to [2], over the last four decades, ungulates such as roe deer (*Capreolus capreolus*), red deer (*Cervus elaphus*) and wild boar (*Sus scrofa*) have dramatically expanded in range in Portugal. Socio-economic changes were the main driver of this expansion: rural exodus with abandonment of agricultural lands, and consequent re-naturalization of the habitats, in addition to more effective laws regarding the creation of protected areas and control of poaching [2], [3]. However, such ungulate expansion can promote changes in ecosystems and can ultimately result in a negative impact *e.g.* on forest regeneration, promote disease transmission and lead to increased traffic collisions [4], [5]. Conversely, ungulates are also a very valuable big game species that generates social and economic income for the rural areas through hunting. Furthermore the role of ungulates as prey for the Iberian-wolf increases its conservation value [2]. Thus, it is crucial to monitor these populations and build effective management plans, supported by reliable wildlife monitoring, to prevent problems arising from the increasing populations and to exploit the potential benefits from such an increase.

A wide variety of techniques have been used to estimate the abundance of ungulate populations (for reviews see [5]–[7]). The selection of the method to implement should take into account the main aim of the study, the logistical and financial resources available, the ecology of the study species and the management questions to be answered [6]. In this study we have used pellet group counts coupled with distance sampling. Distance sampling techniques have been widely used to account for detectability in estimating densities for a variety of taxa such as birds [8], cetacean [9], small mammals [10] and ungulates [11], [12], where pellet group counting is broadly used [13], [14], [15]. Indirect methodologies have been largely applied to a wide range of ecosystems and species, including nests for primates [16], whale blows [17], hare dung [10] and deer [13]. Such methods are based on counting signs produced by the animals [5] and are often referred to as cue counting approaches [18]. Advantages include being easy to implement over large areas, requiring low financial and logistical resources [13] and being especially useful in habitats where animals are difficult to observe directly [6]. These methods follow a two-stage approach, first estimating the density of cues, which is then converted to an estimate of the density of animals by dividing the former by a cue production rate and a cue disappearance rate [18]. Note that these methods therefore avoid the need to assign cues to specific animals.

One of the most abundant and widespread ungulate species in Europe is roe deer [1]. Roe deer's success lies in its ecological and behavioural plasticity that allows adaptations to a variety of habitats [19]. This species density ranges across its European geographical range: while in eastern England it can reach 28.2 ind./100 ha [20], in the Apennine mountains, Italy, it has been estimated a value of 8.5 ind./100 ha [21]. Some studies in the Iberian Peninsula have estimated roe deer density ([22]: 5.56 ind./100 ha), but it is expected that in the edge of its distribution (Portugal), where habitat conditions are theoretically less favourable, population density is likely to be lower [23]. Roe deer is a native species in the north of Portugal, where populations have always persisted [2]. During the 90's a series of reintroductions took place in the centre of Portugal to increase prey availability for the endangered Iberian wolf, *Canis lupus signatus*, and in the south for touristic hunting grounds [24].

Even though roe deer has been widely studied all over Europe [1], [11], [22], [24] the investigation of its ecology in Portugal has recently taken the first steps (*e.g.* [23], [24], [25]). [23] has estimated roe deer density in Montesinho Natural Park and [26] and [27] analyzed the factors affecting this species habitat use, showing that roe deer distribution in northeastern Portugal is positively associated with patches with a high density of shrubs, with increasing distance from roads and negatively associated with spatial heterogeneity. These studies represent a basis for roe deer conservation in Portugal and should be followed-up given that the roe deer population is increasing in the north of Portugal and hunting associations are requesting permission to hunt.

In this study, we aim to determine for the first time roe deer population densities in northeastern Portugal, analyzing data from conventional pellet group counts within the distance sampling framework, while accounting for geographic stratification and the influence of covariates in the detection function. This study will serve as an important baseline for local roe deer long-term monitoring studies and help guide future monitoring efforts and promote game management.

## Methods

### Ethics Statement

Our research did not involve the capture or handling of animals and therefore did not require approval of animal care and use procedures. Permissions for field studies in MNP and Serra da Nogueira were obtained from the Nature and Forestry Conservation Institute.

### Study area

**Figure 1:**
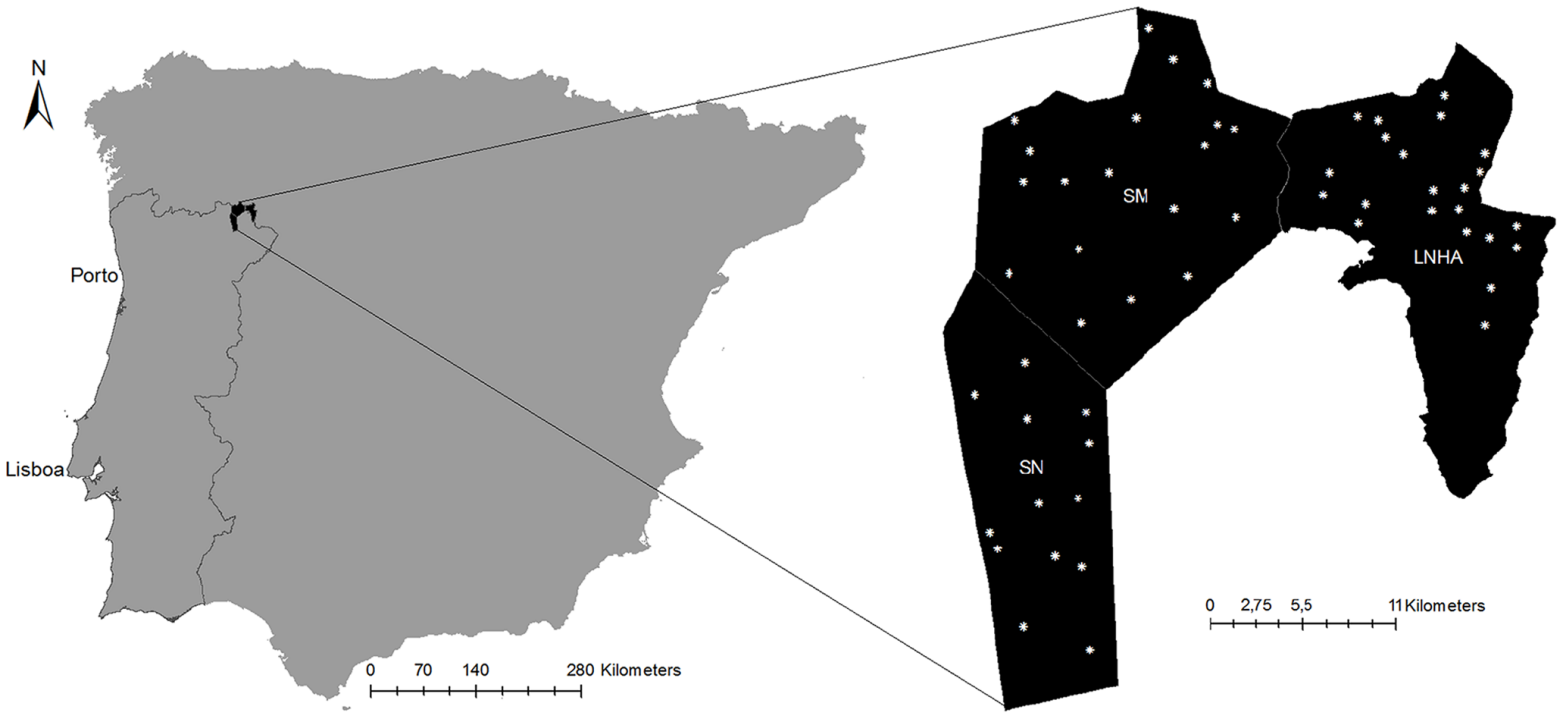
Map of the Iberian Peninsula highlighting where the field work survey was done. Location of the study area in the Iberian Peninsula. On the right there is the distribution of the sampling plots in the three study sites: **SN** – Serra da Nogueira; **SM** – Serra de Montesinho; **LNHA** – Lombada National Hunting Area.

The study was carried out in Montesinho Natural Park and Serra da Nogueira (6°30′–7°12′W, 41°43′–41°59′N and 6°50′–6°56′W, 41°38′–41°48′N, respectively). Both sites are part of the European Union's Natura 2000 Network, covering an area of 63,830 ha (Figure 1). The landscape is mountainous with the highest point located at *Serra de Montesinho* (1,481 m.a.s.l.). The climate is mainly Mediterranean with an annual temperature range between 15°C and 20°C and precipitation varying between 600 mm and 1,500 mm [28]. The vegetation is varied, characterized by oak (*Quercus pyrenaica*, *Quercus rotundifolia*, *Quercus suber*), sweet chestnut (*Castanea sativa*) and maritime pine (*Pinus pinaster*). The shrub vegetation is dominated by heather (*Erica* spp.), gum rockrose (*Cistus ladanifer*) and furze (*Ulex europaeus* and *Ulex minor*). The study area is crossed by some rivers and includes small villages with a low human presence (9.5 people per km^2^).

### Survey design and field methods

Because we expected *a priori* different densities across areas, to improve the precision of a final global density estimate, as well as to provide straightforward separate estimates by relevant management areas, the survey area was divided in 3 geographic strata: *Serra de Montesinho* (SM, 24,800 ha), *Serra da Nogueira* (SN, 18,200 ha) and *Lombada National Hunting Area* (LNHA, 20,830 ha). A total of 54 transects were surveyed. Each survey transect was 1 km long, with 100 m on-effort followed by 200 m off effort, resulting in a total of 600 m off and 400 meters on-effort in each transect. Transect location and orientation were randomly chosen, resulting in 19 transects in SM, 13 transects in SN and 22 transects in LNHA (Figure 1).

Pellet group counts were obtained once from each transect from January 2012 to February 2013. Using a handheld Global Positioning System (GPS) unit and a compass, it was possible to follow a straight line. For practical reasons pellets were only searched in 1 meter vicinity (from both sites) of the transect line, which was defined using a rope laid on the ground to allow for accurate distance measurements. Whenever a pellet group was detected, the perpendicular distance from the centre of each pellet group to the transect line was recorded. To minimize the risk of counting one spread group as two pellet groups [13] we considered only pellet groups with ten or more individual pellets (produced at the same defecation event, identified for similar size, shape, texture and colour) [6]. Red and roe deer pellets can be distinguished through differences in size and shape. Additionally, to account for sources of heterogeneity [29] in the detectability of pellet groups, a number of covariates were recorded: i) dispersion of the pellet-group (aggregated vs. scattered); ii) the type of habitat around the pellet group (open vs. close); iii) the size of the pellet group (medium, between 10 to 40 individual pellets vs. large, more than 40 individual pellets).

### Density estimation

Taking into account species behaviour and habitat conditions, an indirect density estimation method was implemented. Animal density was estimated within a distance sampling [18] framework. Paramount to these methods is the modeling of a detection function, g(*x*), representing the probability of detecting an object of interest given that it is located at perpendicular distance *x* from the transect line. This function can then be used to estimate the detection probability P within the covered area, as
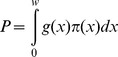
where *w* is a truncation distance and π(x) represents the distribution of available distances. This distribution is assumed to be uniform by design, given the random placement of the transect lines. The estimate of P leads to a density estimator as follows. Given the *n_i_* detected pellet groups in stratum *i*, an animal density estimate is given by
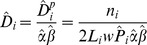
where *L*_i_ represents the total on-effort line length in stratum *i* (*i* = 1,2,3), *P*_i_ represents the detection probability of a group within the covered area in stratum *i*, α represents the production rate: how many pellet groups produces a deer per day; and β the decay of pellet groups: how many days takes a pellet group not to be recognized as a group (> of 6 individuals). Note that the animal density estimator is just the pellet group density (D^p^) estimator, divided by the required production and decay rates. This notation implicitly conveys the assumption that both of these are constant across strata. The global density (D) estimate is obtained as a weighted average of stratum specific estimates, with stratum's areas as weights [18], i.e.
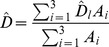



The variance of the stratum specific estimates is obtained via the delta method, by combining the variances of the random components in the estimator defined above (see [18] for details).

In this study the values of α and β were obtained from two different sources. The mean number of days that a pellet group takes to disappear, β, was assumed to be 176 with a SE of ±31 days, a value provided by [30] for roe deer in Montesinho Natural Park. The production rate, α, was considered to be 20 [31], value estimated for UK. We address the plausibility of these values and consequences of bias in these parameters in the final density estimates in the discussion.

The analysis was implemented in software *Distance 6.0* [32]. Multiple Covariate Distance Sampling (MCDS) analyses were used to evaluate the role that covariates can have in the detection function [33] and to assess if a more parsimonious model could be obtained including habitat type, amount of dispersion of pellet group and pellet group size as covariates.

To avoid fitting spurious bumps in the tails of the detection function, data were right-truncated to eliminate 5% of the observations, as recommended by [13], hence discarding observations beyond 90 cm. In the exploratory phase of the analysis the detection function was modelled using half-normal (hn), uniform (u) and hazard-rate (hr) models, combined with series expansion adjustment terms (cosine (c), simple polynomial (sp) and hermite polynomial (hp)) [18]. The most parsimonious model was chosen as that with the lowest Akaike's Information Criterion (AIC) [34], [35]. Chi-squared and Cramer von-Mises goodness-of-fit tests were used as absolute measures of fit to evaluate the adequacy of the final model chosen for inference [for details see section 11.11 in 36].

## Results

**Figure 2:**
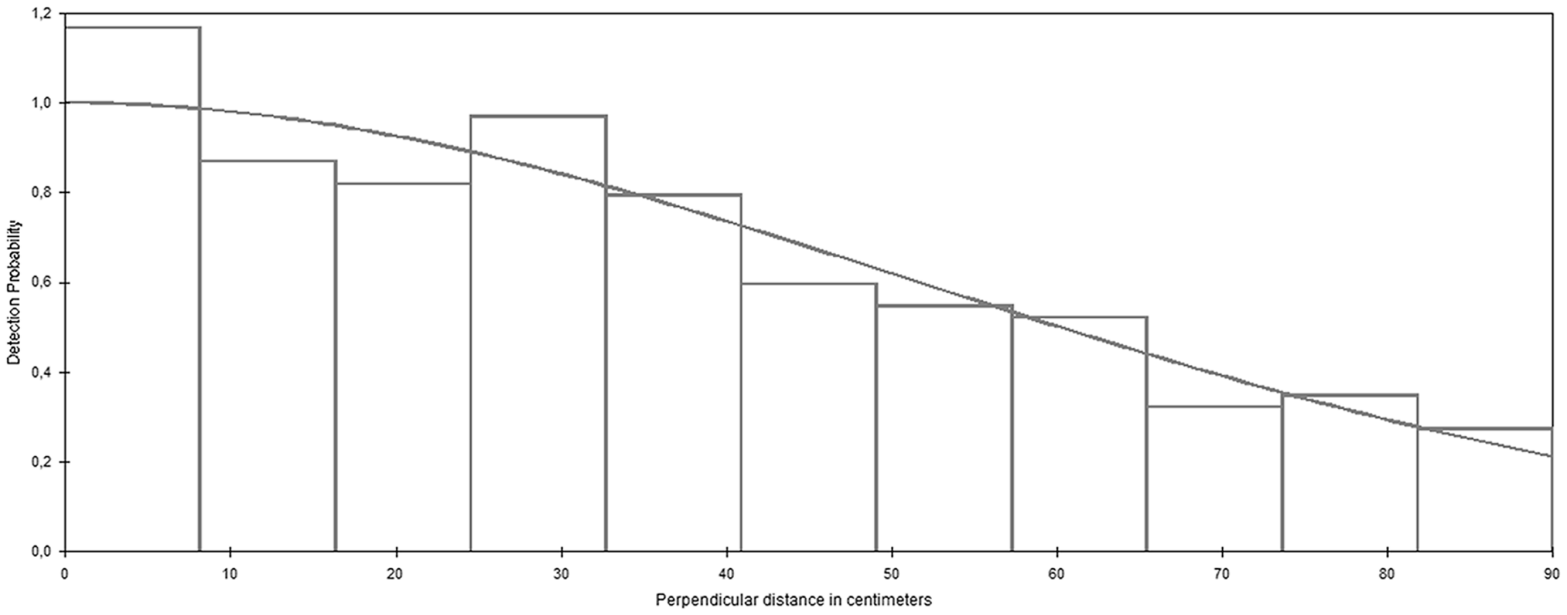
Stratified detection function for the total area. Stratified detection function of the distance data for the survey area using a half-normal key function and a cosine adjustment term. Observed distances were right-truncated to eliminate the largest 5% of the distances. A histogram of the data is superimposed for reference, with the histogram bars scaled such that the area above the model fit is the same as that below. The model was fitted to continuous data, not binned data, and hence the histogram bars cannot be interpreted as probabilities.

In a total of 21,600 m of effort transects (SM with 7,600 m, SN with 5,200 m and LNHA with 8,800 m) 307 pellet groups were recorded. The number of records monotonically decreased with distance (Figure 2), as expected, and no problems were apparent from visual inspection of the data. A half-normal model with a cosine adjustment term (Figure 2) provided the best fit to the data. The best model used a common detection function across the 3 strata. Perhaps surprisingly, none of the covariates contributed to a more parsimonious model, and hence the model with distance alone was selected for further inference. The goodness-of-fit p-values for such model was 0.300 for the Cramer von-Mises test (Table 1) and 0.902 for the chi-squared test. The density estimates per stratum were 1.23/100 ha (95% CI of 0.68 to 2.21) for LNHA, 4.87/100 ha ha (95% CI of 3.08 to 7.71) for SM and 4.25/100 (95% CI of 2.25 to 8.03) for SN and the global density estimate was 3.51/100 ha (95% CI of 2.26 to 5.45) (Table 2).

**Table 1:** Summary statistics for the detection function models considered: AIC, ΔAIC and P-values associated with the χ^2^ and Cramer von-Mises goodness-of-fit (CvM) tests.

Detection function	AIC	ΔAIC	Chi-squared goodness-of-fit	CvM
**Pooled**	2572.67	0.00	0.902	0.300
**Covariate Habitat**	2574.48	1.81	0.025	0.300
**Covariate Size**	2574.17	1.50	0.025	0.300
**Covariate Shape**	2574.10	1.43	0.025	0.300
**Individual – LNHA**	**396.93**		0.363	0.300
**Individual – SM**	**1353.80**		0.630	0.600
**Individual – SN**	**823.10**		0.995	0.800
**Stratified**	2573.83*	1.16		

The “**Stratified**” sumarize the three individual analyses: **LNHA** – Lombada National Hunting Area; **SM** – Serra de Montesinho; **SN** – Serra da Nogueira. Note the χ^2^ outputs of software Distance are based on a smaller number of bins for the CDS analysis than for the MCDS analysis. The results for MCDS might not reliable due to the potential failure of the approximation of the test statistic (see discussion for details).

* This value represents the sum of the three previous individual analyses.

**Table 2:** Roe deer density, abundance and 95% CI estimated using a stratified detection function.

	Area (ha)	Transect length (m)	Total effort (m)	Density (per 100 ha)	Density (95% CI)		Density CV (%)	Abundance	Abundance (95% CI)	
**Total area**	63,830	400	21,600	3.51	2.26	5.45	22.08	2238	1441	3476
**Lombada NHA**	20,830	400	8,800	1.23	0.68	2.21	28.84	256	143	460
**S. Montesinho**	24,800	400	7,600	4.87	3.08	7.71	23.50	1208	763	1912
**S. Nogueira**	18,200	400	5,200	4.25	2.25	8.03	31.76	774	410	1462

Stratified detection function using half-normal model with cosine adjustment term for roe deer estimates for total area and for **Lombada NHA** – Lombada National Hunting Area; **S.Montesinho** – Serra de Montesinho; **S.Nogueira** – Serra da Nogueira.

## Discussion

### Density estimates

The aim of this study was to provide monitoring strategies in order to guarantee a future sustainable exploitation of roe deer without jeopardizing their populations. Higher densities were found in SM and SN. LNHA lower densities can be associated with the presence of the sympatric red deer, which occurs at relatively high densities ([37]: 3.26/100 ha (95% CI - 2.27–4.70) and [38]: 1.75/100 ha (95% CI - 1.07–2.87), thus further investigation is needed to understand interspecific competition between these two ungulates (but for more details see [27]). The obtained coefficient of variation (CV) of density estimation for stratified analysis by area can be considered satisfactory (<31%) according to [39].

[23] has estimated a density of 1–2 ind./100 ha for Montesinho Natural Park (MNP), thus our results suggest an expansion mainly in SM. However it is essential to notice that SN, included in this study, is not part of MNP, hence comparisons should only be made taking into account LNHA and SM. Nonetheless, in an European [20], [40], and even in an Iberian [22] context our results correspond to minimal values of density.

### Method assumptions

Here we used an indirect method based on pellet groups to estimate roe deer density. This is a distance sampling based approach and therefore based on the usual distance sampling assumptions. The distribution of available distances within the covered area is assumed to be uniform and this is usually enforced by design. Distance sampling is based on four key assumptions: (1) objects (pellet groups in this case) on the transect line are always detected. It is unlikely that the pellet groups lying on the line are missed, but even if they were, given that we are looking for static objects in a very narrow transect, the g(0) = 1 assumption would suffer at worst minor violations; (2) sampling is instantaneous, in practice requiring that animals move slowly compared to observers and especially that animals do not move in response to observer before being detected. Because pellets are immobile this assumption holds with certainty; (3) perpendicular distances to the centre of the transect line are accurate [29], [35]. The field methods used ensured that any violation of the measurement error assumption would be minor, certainly within the realms of what is negligible in practice (*e.g.* [41]); Obtaining estimates for the parameters of the detection function by maximum likelihood requires that (4) detection events are assumed independent, but methods are very robust to the failure of this assumption.

The p-value of the chi-squared test reported by default in software Distance for the model used for inference was 0.032, which could be taken as evidence of a sub-optimal fit, unlike the value of 0.902 reported in Table 1. This is due to the distance bins used in the estimate reported by default by Distance, which considers the largest number of bins from 3 sets attempted (this is the only value reported by the software from an MCDS analysis). For our data set, under that scenario there are not enough observations per bin, hence the chi-squared approximation is inadequate. This clearly shows how care should be taken in the interpretation of the chi-squared test. Here, and in general with continuous data, the CvM is more reliable and does not depend on the binning used. Hence we conclude that the fit was adequate, as is apparent in Figure 2. The number of transects used was about 4 to 5 times larger than the usual recommendation (*e.g.* [18]), and therefore variance estimates should be robust. The estimates are also representative of the wider survey region and the uniform assumption is likely to hold. Despite being more labour intensive, the sampling design was tailored to achieve more accurate estimates (*e.g.* through the use of random transects), which does not occur regularly in ungulate studies (*e.g.* [12], [42]). This can lead to bias if sampling is not well planned (*e.g.* [41]).

The decay rate used is for the species and region of interest, however, this value refers to pellet groups with six or more individuals, while in our survey only groups with ten or more pellets were recorded. When a pellet group is defined as 10 individuals less bias is expected, since there is less chance of misclassifying large and relatively dispersed pellet groups as two or more independent groups. Therefore we chose to ignore pellet groups with fewer than ten individual pellets. Since disappearance days can vary among habitats, the use of a site specific value for each dominant habitat in each place (as estimated by [30]), over the mean value, should be assessed in future works. [43] has estimated a decay rate of 220±20 days for roe deer in Scotland, which, if used, would result in lower density estimates. However, since we have a site-specific value of disappearance days we chose to use [30] over [43].

The key problem with our estimate is related to the use of a production rate obtained for another place and time, namely the UK in the 2000's [31]. Furthermore, the value used does not have a variance or standard error associated, which means that the reported variance of density estimates ignores a potential source of variation. However, a clear advantage of the modular form of the estimator used is that, as soon as a production rate and corresponding standard error are obtained for this region, the density estimates and corresponding variances reported here could be easily updated. Obtaining such production rate should therefore be a major goal for the effective management of these populations. If we can assume that the production rate is spatially and temporally constant, density comparisons over time and space are insensitive to this parameter. Changes in α and β necessarily lead to different density estimates, as the deer density estimate is just the pellet group density estimator divided by production and decay rates. This necessarily implies that an increase in either factor would result in a lower density estimate, and vice versa. As an example, given our results a deviation of 10% would have a minor impact in the estimates: the density for α+10% would become 3.19 ind./100 ha, while for α−10% it would become 3.90 ind./100 ha. While for β+10% would become 3.18 ind./100 ha and for β−10% it would become 3.91 ind./100 ha.

Perhaps surprisingly, as we had selected only covariates *a priori* thought to influence detectability, no covariate was considered important in modelling detectability beside distance itself. This reflects the fact that distance sampling pooling robustness property is strong and that sometimes MCDS provides no additional practical gain beyond conventional distance sampling. Nonetheless, under certain circumstances MCDS can be used to reduce variance estimates, by explaining some of the variance in detectability. MCDS might also allow less biased estimates of density. In fact covariate influence on detectability might be interesting in itself. Therefore, our recommendation is still that covariates likely to affect detectability should be collected and tested for possible inclusion in the analysis.

### Methodology

Estimation of densities can be controversial due to its reliability and to the choice of the suitable method for each case. According to [44], three questions should be answered before a monitoring program begins: (a) why monitor? – Management or scientific purposes; (b) what should be monitored? – Assess which species should preferentially be monitored due, for example, to a rapid increase or species with socio-economic benefits underexploited such as ungulates; and (c) how should monitoring be carried out? – Evaluating aspects such as the relation of effort-survey area, randomly placed transects and stratification among areas as base criteria for designing surveys [29]. The choice of the method for this study appears to be reasonable. However some authors [5] argued against pellet group counts due to its high variance leading to wide 95% confidence intervals in density estimates, making them of low informative value and practical use, although bias due to violation of distance sampling assumptions is likely to be negligible when applying this methodology. [7] has also argued against indirect methods due to their inability to provide data beside population size in itself, which they believe has no informative value with regard to demographic fluctuations. In fact, [7] stated that in areas with low visibility, hunting-related methods are frequently used. However, hunting habits in our study area are not very frequent. Considering this and given species behaviour, the prevalence of concealing areas, the need for roe deer density estimates in our study area, and logistical constraints, indirect methodologies seemed to be the adequate approach. Other authors have recommended this methodology, arguing that it can determine population size and trends and can be used for conservation purposes [12]. Furthermore, the method is simple and cheaper than other approaches, can act as an indicator of geographic distribution and the results have proved to be reliable elsewhere [13], [11], [45], [46]. In our study, we coupled pellet group counts with the widely used distance sampling approach [11], [12], [15], [40]. [47] stated that when considering indirect approaches, line transect distance sampling is the most efficient method to obtain ungulate density estimates.

### Future work and general management recommendations

Indirect methods based on pellet group counts have some drawbacks, for example they do not provide information on age class distribution, sex ratios and productivity. Conversely, pellet group counts have the advantage of providing estimates that integrate a broader time span with relatively low effort in terms of time, labour and logistical resources, providing a favourable balance between cost and performance in practice [48].

Even though this methodology addresses the requirements for management purposes, provides an estimate of the size of the population and allows the following of trends. Future research efforts should have to attempt to apply other methodologies to monitor roe deer populations, with a positive balance between cost and performance. The potential benefits of direct animal-based rather than pellet-based distance sampling methods to estimate roe deer density in this area should be assessed. Nevertheless, this study fills a gap in conservation and management of roe deer in northeastern Portugal, through the provision of density estimates, although more information is needed to implement a conservation plan based on continuous scientific knowledge.

In fact, collecting data for estimating densities is essential for the effective monitoring of populations. We are hopeful that in coming years the collaboration of hunters and rangers will facilitate data collection. The roe deer population monitoring should continue since long-term survey data is required to assess the impact of management practices. Monitoring programs should include an assessment of deer abundance and their impacts on agriculture, forestry and vegetation.
